# *On the* Formation *of* Fat *in the* Intestines *of* Living Animals

**Published:** 1814-01

**Authors:** Everard Home

**Affiliations:** Bart.


*For the Medical and Physical Journal.*

### *On the* FORMATION *of* FAT *in the* INTESTINES *of* LIVING ANIMALS; *by* SIR EVERARD HOME, BART.

THE investigation of the digestive organs of different animals, in which I have been engaged for many years, has led me imperceptibly into an inquiry respecting the particular uses of the lower portion of the intestines in birds and quadrupeds.

The first thing that attracted my notice more particularly to this subject, was finding that in all animals, whose stomachs are made up of a great variety of parts for the purpose of economizing the food, the colon has a greater extent of surface, and the course of the canal is so disposed, that its contents must be a long time in their passage through it. This circumstance led me to believe that the food, after the chyle is formed and separated from it, undergoes in the lower intestines some changes, by which a secondary kind of nourishment is extracted from it.

The opinion was much strengthened, by finding that the colon of the casuary from Java is only one foot long, and each of the cæca, which are appendages of it, only six inches long, and a quarter of an inch in diameter; while the African ostrich has the colon forty-five feet, and each of the cæca two feet nine inches in length, and at the widest part three inches in diameter: besides which, both the colon and cæca have very broad valvulæ conniventes not met with in the casuary from Java. This wonderful difference, for it is more than fifty to one, can only be explained by the luxuriancy of Java being so great, that this bird might destroy its health by over feeding, had no guard been furnished by nature.

This guard is, the food passing through the intestines with so much facility, and in so short a time, that however much the bird may eat, only the necessary quantity of nourishment is carried into the constitution; but in the African ostrich, the food is retained in the extensive colon till every thing nutritious is extracted. In all ruminating animals, the colon is of great length, is fixed in its course, which is very intricate, and varies in every different genus; so that we cannot doubt of some particular process being carried on in it.

The process which the contents of the colon undergo, is quite distinct from any thing carried on in the other intestines, since they intirely change their appearance and smell; and there is commonly a valve to prevent any part of them, even the gases evolved, from being carried up into the small intestines.

 The

The peculiar smell of the fæces, which borders so closely on that from putrefaction, although by no means the same, led me to compare them with the animal matter buried in the earth, which is converted into adipocere: in both cases the substance is in the incipient state of putrefaction, but that process never completely takes place; it is excluded from the external air, is either under water or within the reach of imbibing moisture; and there is no substance whatever, the chyle excepted, which can better supply the waste produced by the actions of growth and muscular exertion, than animal fat.

The more I canvassed this new opinion, the greater number of circumstances in favor of it occurred to me; one of the strongest of which is, that there is no other mode I am acquainted with, by which animal fat can be formed. To this may be added the curious circumstance of the sleeping animals, which lay in so large a supply of it, in a short time, to serve for their winter's consumption, having a formation of the intestines almost peculiar to themselves, in which there is no valve to distinguish the colon, and no fixed course for that intestine; so that the contents pass along with more facility, and remain a shorter time in the canal, the food being sufficiently plentiful during the summer to compensate for this want of economy, by which the lower intestines receive more abundant supplies for the production of fat. These intestines remain empty during the sleeping season, so that no fat can be formed in that period.—With this very important information, thus procured, in support of my opinion, I have been led to prosecute this inquiry with increased ardour, and shall now bring forward the facts I have been able to ascertain in confirmation of my hypothesis. These I shall detail in the order in which they were acquired, thinking it better to lay before the society the regular process of the investigation, than to grasp at once at the conclusions, which in the end of it I have felt myself authorised to draw.

I shall therefore begin by stating the circumstances under which adipocere is formed from animal matter, most nearly resembling those in which the contents of the lower intestines in living animals are placed; and this I shall do from facts, entirely within my own knowledge, the specimens of the adipocere being now in my possession; and afterwards go on by bringing forward proofs that a substance similar to it is formed in the colon.

Mary Howard, aged forty-four, died on the 12th of May, 1790, and was buried in a grave ten feet deep at the east end of Shoreditch church-yard, ten feet to the east of the great common sewer, which runs north and south, and has always always a current of water in it, the usual level of which is eight feet below the surface of the ground, and two feet above the level of the coffins in the graves. In August, 1811, the body was taken up with some others buried near it, for the purpose of building a vault, and the flesh in all of them was completely converted into adipocere or spermaceti. In Stow's History of London, this part of Shoreditch is stated to be a morass, and since that time the ground has been raised eight feet. The clerk and the grave-digger observe, that at the full and new moon the water in the sewer rises two feet, and that at those times, there is water found in the graves, which at other times are dry.

The current of water, which passes through the colon, while the loculated lateral parts are full of solid matter, places the solid contents in somewhat similar circumstances to dead bodies on the banks of a common sewer.

The circumstance of ambergris, which contains sixty per cent. of fat, being found, in immense quantities, in the lower intestines of the spermaceti whales, and never higher up than seven feet from the anus, is an undeniable proof of fat being formed in the intestines; and, as the ambergris is only met with in whales out of health, it is most probably collected there from the absorbents under the influence of disease, not acting so as to take it into the constitution.

Ambergris is found in lumps from fourteen to more than one hundred pounds each; it is not to be distinguished in its appearance from the fæces, but when exposed to the air, it grows hard: a lump has been found in the sea weighing one hundred and eighty-two pounds.*

In the human colon, solid masses of fat are sometimes met with in a diseased state of that canal, and are called *scybala;* these are in all respects similar to ambergris.

Concretions of olive oil and mucus found in the human intestines must be formed in the same way. A case of this kind was communicated to me by our associate Dr. Babington, in the following letter:

"My dear Sir, 17, *Aldermanbury, Feb.* 2, 1813.

"The following are the circumstances relating to the change produced upon olive oil, by passing through the stomach and intestines of the elderly person, whose case I mentioned to you at the last meeting of our Animal Chemistry Society. The lady, in question, had for several years past suffered from severe affections of the stomach, which, from the attendant symptoms, were considered as occasioned by the irritations of biliary concretions. Many remedies having been resorted to without affording her other than temporary benefit, she

* Vide Phil. Trans. 1783.

was was advised to try the effects of olive oil, taken to the quantity of two or three ounces at a time, and to be repeated as circumstances might require. From this she experienced almost immediate relief, and, on the subsequent examination of what passed from the bowels, globular concretions were uniformly observed, which by the persons about her were considered as the gall-stones, which had previously been productive of so much distress. This lady having occasion some months since to visit her friends in town, and a doubt having been suggested by one of her medical attendants in the country, as to the nature of the concretions in question, I was desirous, from the account that I had received, to have an opportunity of determining the point for myself; and therefore requested, that if the pain should recur, and she should be under the necessity of repeating her medicine, that the concretions, which had been said always to pass from the bowels in consequence of her so doing, might be reserved for my inspection. In a few days I was summoned to make my proposed visit, and, upon examining the substances collected, I found their appearance to be such as I have already described to you, namely, that of distinct globules, varying in size from that of a large pea to the bulk of a moderate grape, of a cream color, and slightly translucent, of sufficient consistence to preserve their form, and to bear being cut by a knife, like soft wax, but at the points of their contact disposed to cohere. When exposed to heat, they readily melted, and then at once exhibited their original oily character. The change, which they have since experienced, has taken place in the water in which they have been kept. I am, dear Sir,

"Your's always, very faithfully,

(Signed) "WM. BABINGTON."

"*To Sir Everard Home, Bart.*"

Our associate, Mr. Brande, afterwards examined the substance, and made the following report upon it:

"The globules voided appeared to be composed of the olive oil combined with mucus: the latter separated during putrefaction, and the oil was evolved, apparently unaltered. The relative proportion may be estimated at one-third animal matter, and two-thirds vegetable oil."

The following case, which was also communicated to me by Dr. Babington, shows that fat is sometimes formed in the intestines, and detected passing off with the fæces.

Elizabeth Ryder, four years and a half old, had been healthy for six months after her birth, when she became thin, had a sallow complexion, and was liable to jaundice. At a year and a half old, her belly was tumid, and she had great weakness in her back and limbs, for which complaints Dr. Babington was first consulted. At three years old, her mother observed something come from her, as she walked across the room, which, when examined, was found to be fat in a liquid state, which concreted when cold. Ever since that time to the present, she has voided, at intervals of ten or fourteen fourteen days, the quantity of from one to three ounces, sometimes pure, at others mixed with fæces; when voided, it has an unusually yellow tinge, and is quite fluid like oil. Her appetite is good, as well as her spirits, and her flesh firm; her belly rather tumid, but not hard: she is subject to occasional griping: her urine natural, and she sleeps well. The specimen on the table was procured under circumstances which precluded all possibility of deception.

These facts, so strongly in favor of the opinion I had taken up, led me to devise in what way it might be put to the test of experiment. I tried to extract fat from the contents of the colon in different parts of its course, but without success. Failing in this mode of obtaining any decisive conclusions, I was led to believe the cæca of birds more favorable for experiments on this subject, and had those of a duck examined by Mr. W. Brande after the bird had been seven days without an evacuation. This confined state of the bowels put the parts nearly under the same circumstances, as if they were in a diseased state. When the cæca were examined, they were found completely distended with fæces of the consistence of soft clay, so that, when the bags were laid open, the contents retained the same form. The intestine, immediately above the cæca, was empty, but the rectum was much distended: its contents were of a softer consistence than those of the cæca.

The following is Mr. Brande's report on this subject:

" The contents of the cæca were divided into two portions, of one drachm each, and comparative experiments were made with similar quantities of the contents of the rectum.

" *Exp.* 1.—One drachm of the contents of the cæcum was completely immersed in half an ounce of water, and kept for seven days in a temperature varying from 40° to 60°. At the end of that time, warm water was poured upon it, but no appearance of fat could be perceived.

" *Exp.* 2.—The same quantity of the contents of the cæcum was immersed in water containing one-fifteenth part of nitric acid, and kept under the same circumstances as in the former experiment. In seven days, warm water poured upon it separated a portion of oily matter, which concreted when cold, and appeared to be one-eighth of the whole mass.

" *Exp.* 3.—Portions of the contents of the rectum were treated in the same way as in experiments 1 and 2. That in water became putrid very rapidly, and showed no appearance of fat. The other, in the diluted nitric acid, was more dissolved than in experiment 2. Considerable extrications of gas took place, but there was no appearance of fat."

From these experiments, we learn that the contents of the cæcum, being confined there for some days, are in a state readily to be converted into fat by nitric acid, while the contents of the rectum are not, probably from being too putrid.

While engaged in this inquiry, I received from Sir Joseph Banks the carcase of a wild swan, which the Hon. Mr. Pelham had shot in the neighbourhood of the Humber. On examining the cæca, their contents were found to be of a bright green color. This led me to propose to Mr. Brande to ascertain by experiment, whether an admixture of bile had any effect upon the process of converting animal substance into fat. The following experiments were made by Mr. Brande upon this subject.

*Exp.* 1.—He took two portions of human muscle of the same size, and digested one of them in human bile, the other in water, both placed in the temperature of 100°. In the first day the muscle in the bile underwent no change. On the second day it became soft in its texture, and had a slightly fetid smell. On the third day it became more fetid and yellow. On the fourth it had the smell of excrement, was flabby, very offensive, and fatty upon the surface. The portion of muscle, digested in water, had undergone no other change in the four days but becoming slightly putrid, and there was no appearance of fat whatever.

*Exp.* 2.—A similar experiment to that with the human bile, was made with a small portion of beef, and ox's bile, and the results were exactly similar.

*Exp* 3.—The last experiment was repeated in the temperature of 60°. In four days the beef became slightly fetid, and of a yellow color; in six days it became more fetid, but there was no appearance of fatty matter.

*Exp.* 4.—A portion of beef cut into pieces was digested in ox's bile, at the temperature of 100°. At the end of the fourth day the putrefaction was more advanced than in experiment 2: the beef was washed and heated upon paper, but no greasy stain was produced.

From these experiments we learn, that the bile has a power of converting animal substance into fat; and that the temperature of 100°, or nearly so, is necessary for that process. We learn also, that this change is produced just as putrefaction is beginning to take place, and if the substance goes rapidly into putrefaction, no fat is formed, and, what is deserving of observation, the peculiar smell belonging to fæces, so so different from that of putrid matter, is produced at the time that fat is procured.

Having succeeded in changing animal matter into fat, by adding bile to it out of the body, I was desirous of ascertaining whether this process could be detected going on in the human intestines; and being in attendance upon a gentleman of an advanced age, who had been six days without an evacuation from the bowels, confined to bed by the gout, I did not let slip the opportunity of his having a very costive stool deeply tinged with bile, to make the experiment. The excrement was put into water, and kept heated for three hours to a temperature of above 100°. When the water was allowed to cool, a film was formed upon the surface, which appeared to be of an oily nature, and Mr. Brande ascertained it to be so. The quantity was not great, but quite sufficient to ascertain the fact; and next day the fæces having subsided, the fatty film was much more conspicuous. In the Phil. Trans. for 1673, p. 6093, a case is stated of a person who labored under an indisposition, attended with sickness and vomiting. In one attack of vomiting, he brought up matter resembling tallow, four pieces of which weighed half an ounce.

This process of forming fat in the lower intestines by means of bile, throws considerable light upon the nourishment derived from clysters, a fact well ascertained, but which could not be explained. It also accounts for the wasting of the body, which so invariably attends upon all complaints of the lower bowels. It accounts, too, for all the varieties in the turns of the colon, which we meet with in so great a degree in different animals. This property of the bile explains likewise the formation of fatty concretions in the gall-bladder, so commonly met with, and which, from these experiments, appear to be produced by the action of the bile on the mucus secreted in the gall-bladder; and it enables us to understand the following effects, which arose from the circumstance of no part of the bile passing into the intestines.

A child was born, at the full time, of the usual size, and lived for several months, but never appeared to increase in size, although it fed heartily, had regular stools, and the food seemed perfectly digested. There was no bile in the stools, and the skin was of a dark yellowish brown. I saw the child while it was alive, and was struck with its want of growth, and its having no fat under the skin, which made it appear longer than new-born children generally are. Upon examining the body after death, the only mal-formation met with, was there being no gall-bladder, nor any duct leading from the liver into the duodenum.

From what happened in this case, a supply of fat appears necessary for growth, for the child was by no means wasted in its muscles, which it must have been had the constitution not been supplied with nourishment.

Animal fat has, I believe, hitherto been considered as a secretion, although there is no direct evidence in favor of such an opinion. It has nothing in common with the secretions; it is met with in all the interstices of the body; is very often quickly deposited, and in as short a time taken back into the constitution. In these respects it corresponds with the watery fluids, with which the body is supplied.

In a former communication respecting the stomachs of animals, I explained that water was taken up from the stomach by channels yet unknown, and carried into the circulation, from whence it is poured into all the cavities of the body, or thrown out altogether by the kidneys and glands of the skin.

On the present occasion, I hope that I have collected a sufficient body of evidence to prove that fat is formed in the intestines, and from thence received into the circulation, and deposited in almost every part of the body. When there is a great demand for it, as in youth, for carrying on the growth of the body, it is laid immediately under the skin, or in the neighbourhood of the abdomen: when not likely to be wanted, as in old age, it is deposited in the interstices of muscles, to make up in bulk for the wasting of the muscular fibres. There appear to be no direct channels by which any superabundance of it can be thrown out of the body, so that when the supply exceeds the consumption, its accumulation becomes a disease, and frequently a very distressing one.—*Phil. Trans. Part II. for* 1813.

